# The Mechanism of Action and Experimental Verification of Narenmandula in the Treatment of Postmenopausal Osteoporosis

**DOI:** 10.2174/0113862073264965231116105323

**Published:** 2024-01-04

**Authors:** Jirimutu Xiao, Ziceng Yu, Qiuge Han, Yang Guo, Jiapeng Ye, Hua Lian, Lining Wang, Yong Ma, Mengmin Liu

**Affiliations:** 1 Laboratory of New Techniques of Restoration & Reconstruction of Orthopedics and Traumatology, Nanjing University of Chinese Medicine, Nanjing, China;; 2 Inner Mongolia Medical University, Inner Mongolia, Hohhot, China;; 3 School of Chinese Medicine, School of Integrated Chinese and Western Medicine, Nanjing University of Chinese Medicine, Nanjing, China

**Keywords:** Postmenopausal osteoporosis, narenmandula, mongolian medicine, oophorectomy, network pharmacology, molecular docking

## Abstract

**Background:**

Narenmandula is a classic ancient remedy in Inner Mongolia, historically used for gastrointestinal diseases. In recent decades, Inner Mongolia Medical University found that it has a significant effect in promoting fracture healing and increasing bone density, and has been used to treat postmenopausal osteoporosis (PMOP), but its mechanism is unclear.

**Objective:**

Identify the mechanism of action of Narenmandula for PMOP treatment.

**Methods:**

Network pharmacology, molecular docking and ovarian departing rat models were used to verify the relevant mechanism of Narenmandula in the treatment of PMOP.

**Results:**

We confirmed that NRMDL prescription can improve OVX-induced bone loss, improve trabecular density, and relieve osteoporosis. Upon screening of network pharmacology, we obtained 238 overlapping genes of Narenmandula and PMOP, and analyzed AKT, IL1B, and IL6 as key genes by network topology. Among the 1143 target genes that interact with PMOP, 107 NRMDL active compounds correspond to 345 target genes and 238 overlapping genes. Network topology analysis showed the top 8 active ingredients, such as quercetin and kaempferol, and the top 20 key genes, such as AKT, IL1B, IL6, INS, JUN, STAT3, TNF, TP53, *etc*. Enrichment analysis revealed involvement of PI3K-Akt, HIF-1, FoxO, MAPK, and TNF signaling pathways. In addition, we found the most important active compounds bind tightly to core proteins, which were verified by molecular docking analysis. The AKT-related pathway had good binding energy, and the pathway was verified by cell and animal experiments.

**Conclusion:**

The potential mechanism and efficacy of Narenmandula against PMOP may be related to the PI3K-AKT pathway.

## 1 INTRODUCTION

Postmenopausal osteoporosis (PMOP) is a common disease related to aging, mainly occurring in postmenopausal women. Severe estrogen deficiency causes increased osteoclastic activity, decreased bone density, increased bone conversion rate, changes in calcium salt deposition, increased bone ablation, bone loss, and eventually leads to PMOP. Common clinical signs include changes in bone tissue structure, increased bone fragility, risk of fractures, as well as pain caused by fracture, and bone deformation seriously affect the physical health and quality of life of the elderly, and even shorten life span. The prevalence of osteoporosis in postmenopausal women is four times that of men. Early PMOP is characterized by rapid bone loss; late PMOP occurs 10–20 years after menopause with slower bone loss, although secondary hyperparathyroidism worsens PMOP. Therefore, as estrogen levels decline, the incidence of osteoporosis in postmenopausal women rises dramatically. However, osteoporosis is an insidious disease that often has no symptoms until a fracture occurs, and upon detection of a hunchback, shortness or bone pain. Studies have shown that hormone replacement therapy can increase estrogen levels, reduce bone turnover, inhibit bone resorption, and balance bone metabolism, but its safety is still controversial. As a first-line treatment for PMOP, bisphosphonates have a highly effective ability to inhibit bone resorption, however, their long-term effects on renal function and the risk of mandibular lysis remain to be investigated. Therefore, there is an urgent need for alternative drugs in clinical practice. Mongolian medicine, deeply trusted by people as a part of the medicine of the motherland, has unique advantages in the treatment of postmenopausal osteoporosis with few side effects and good efficacy.

Mongolian medicine has its unique experience and efficacy in the prevention and treatment of osteoporosis. Mongolian medicine places osteoporosis in the category of “bone Heyi disease and bone blight”. The reason is that the imbalance of the three “Heyi, Sheila, and Badagan” and the turbid metabolism of the seven elements “food essence, blood, muscle, fat, bone, bone marrow, and essence”, resulting in a decrease in bone strength and the formation of bone blight [1]. Mongolian medicine Narenmandula (NRMDL), pulled out from “Supreme Essentials”, is one of the commonly used compound remedies in Inner Mongolia, composed of pomegranate, cinnamon, nootropic kernel, cubeb, safflower, Xiaoshu ji, jade bamboo, huangjing, tianmen dong, *Tribulus terrestris*, smallpox powder, has been included in the “Ministry of Health of the People's Republic of China Drug Standards - Mongolian Medicine Volume”. It mainly has the effects of enhancing stomach fire, promoting digestion, tonifying kidney and aphrodisiac, promoting turbidity metabolism, inhibiting bone withering, and Heyi disease [2]. Clinical studies have found that NRMDL can relieve pain caused by osteoporosis and increase bone mass, but its mechanism of action is not fully understood.

Network pharmacology, a concept first proposed by Andrew L Hopkins in 2007, has become one of the frontiers and hot spots in the field of traditional Chinese medicine research in recent years. The systematic regulation and synergy of herbal compounds have special advantages in disease prevention and control, but herbal compounds are rich in pharmacodynamic substances, have a complex mechanism of action, pose difficulty to control the quality of medicinal materials, and challenging to study from general properties to the molecular level. Therefore, it is important to establish a holistic research protocol that is consistent with herbal remedies for disease prevention. Network pharmacology starts from the overall model and predicts relevant signaling pathways through pharmacodynamic molecules. In addition, molecular docking simulations are used to detect binding to verify the stability of key proteins and pharmacodynamic molecules. In this paper, network pharmacology, molecular docking and ovarian departing rat models were used to verify the relevant mechanism of NRMDL in the treatment of PMOP, and further explore its mechanism of action.

## 2 MATERIALS AND METHODS

### 2.1 Animal Experiments

#### 2.1.1 NRMDL Preparation

Composition of NRMDL is found in Table **1**.

#### 2.1.2 Animal Model Preparation

A total of 48 two-month-old female rats with SPF-level SD were weighed (250±15 g), animal qualification certificate: SCXK (Zhejiang) 1019-0002 (approved by the Animal Ethics Committee of Nanjing University of Chinese Medicine: A200904). Each group of four rats was housed in one cage with a light time of 12 h/d, temperature of 25°C, ventilation and drying, and free diet. Sham surgery (sham, n=8) and bilateral oophorectomy (OVX, n=40) were performed respectively after one week of acclimation (all animal procedures comply with international ethical guidelines and National Health Institute guidelines for laboratory animal care and use). We try to reduce the number and suffering of animals; all surgeries use air anesthesia machines (Anesthetics: isoflurane; Flow rate: 0.5~0.7L/min) and aseptic techniques. After surgery, erythromycin ointment is applied to the wound to fight infection. If the bone density of rats decreased significantly three months after removal of ovaries, the wound of the OVX animal model was considered successful.

#### 2.1.3 Extraction and Culture of Osteoblasts

Five 1-day-old SPF-grade SD milk rats were weighed (10±5 g), animal qualification certificate: SCXK (Shanghai) 2018-0004, provided by Qinglongshan Animal Experiment Center, Jiangning District, Nanjing. The skull was soaked in 75% ethanol for 5 min and removed under sterile conditions, the fascial tissue was removed in a PBS solution of 3% bispecific antibody, the bone pieces were sheared and digested in a 0.25% pancreatic incubator for 30 min, the digestion was terminated with the whole companion. Supernatant was centrifuged (1000 r/5min) and 0.1% type I collagenase was added, then incubated in constant temperature shaking box (37°C, 80 r/h) for 4 h, the bone fragments were removed by 70 μm filter and centrifuged (1000 r/5 min) to go to the supernatant, and rinsed with PBS. The whole culture was inoculated in a T25 flask; when the cell confluency reached about 90%, 2.5% pancrepsinization was digested for 1.5 min, and then the digestion was terminated by full accompaniment, centrifugation (1000 r/5 min), supernatant was added to the full companion passage, and the 3rd generation cells were taken for subsequent experiments.

#### 2.1.4 Pharmacological Interventions

The successful rat models were randomly divided into 5 groups: model group (OVX), model group + NRMDL pill low dose (Low), model group + NRMDL medium dose (Middle), model group + NRMDL high dose (High), model group + bone peptide tablets (OT). Sham and model groups were given distilled water by gavage, the model group + NRMDL low/medium/high dose groups were given 0.2 mg/(kg•d), 0.4 mg/(kg•d), 0.8 mg/(kg•d), respectively, and the model group + bone peptide sheet group 90 mg/(kg•d) by gavage for 12 weeks. The intake dose of rats was carried out according to half times, double and twice the clinical human dose, and the conversion followed the principle of human mouse equivalent dose conversion. After 12 weeks, rats were anesthetized using a gas anesthesia machine following the last 24 hours of drug treatment. Abdominal aortic blood was centrifuged at 3500 rpm for 10 min, serum was collected, and stored at -20°C. Specimens were taken from the lumbar vertebral body, bilateral femur and tibia.

Drug-containing serum was prepared according to the experimental dose of animals: low dose of Mongolian medicine (NL) was 0.4mg/(kg•d), 2mL per day; Mongolian high-dose group (NH) was 0.8 mg/(kg•d), 2 mL per day; and blank group given 2 mL saline . A total of three animals from each group of four were administered treatments by gavage continuously for three days, anesthetized by gas anesthesia machine two hours after the last gavage. Blood was taken from the abdominal aorta, centrifuged (3500 r/min, 10 min) after standing at 4°C for 2 hours, the upper serum taken, sterilized with a 0.22 um membrane, and stored at -80°C.

#### 2.1.5 Enzyme-linked Immunosorbent Assay (ELISA) Test

ELISA detection reagent (Nanjing Jiancheng Co., Ltd.) was used to detect the content of TRPA and ALP in serum. According to the instructions of the kit, set negative and positive controls (2 wells each), add samples (50 μL / well), add microplate label secondary antibodies (50 μL/ well) and antibody working solution (100 μL / well), cover plate and place in a 37°C light-protected incubator. Wash the plate 4 times, add substrate (100 μL/ well), cover plate, incubate at 37°C to avoid light for 10 min, and immediately after adding the stop solution, the absorbance OD value of each reaction well at a wavelength of 450 nm was determined with a microplate reader.

#### 2.1.6 Polarographic Detection of Serum Trace Elements

Serum trace element detector (China Sankai Medical Technology Co., Ltd.) detects zinc, phosphorus, sodium, calcium, magnesium and other trace elements in serum. The machine collects serum (100ul per sample) and the peak current detected is quantitatively analyzed according to Ip= KC (Ip is the peak current, K is the constant, and C is the concentration of the substance to be measured).

#### 2.1.7 Micro-CT Detects Changes in the Microstructure of the Femur

From each group, five rat right femur samples were randomly selected and micro-CT (Bruker, SKYSCAN 1272) was used to observe the bone structure and mineral changes. A micro-CT imaging system (Analyse12) was applied for CT scanning and trabecular morphological analysis with the following parameters: spatial resolution of 9 μm (X-ray source 80 kV, 384 μA); scan right femur specimen with a 1 mm filter. The CT images are reconstructed with the built-in software CT-vox and CTAn, respectively. With the recommended sequence set, CTAn selects trabecular regions of interest in unbiased batches. The purpose of trabecular analysis is to quantify morphological calculations and bone density. The direct measurement parameters for measuring the right femoral trabecular are: trabecular thickness (Tb.Th), bone volume fraction (BV/TV), number of trabecular bone (Tb.N), and trabecular spacing (Tb.Sp).

#### 2.1.8 HE Staining to Observe Morphological Changes in the Femur

The distal end of the right femur is placed in 4% paraformaldehyde fixation for 48 h, rinsed with PBS buffer thrice for 20 min, placed into EDTA decalcification solution, placed in a 37°C incubator for 4 weeks until the pinus can puncture the femur and stop the decalcification. Subsequently, dehydration, transparency, embedding, sectioning and other steps are carried out. After HE staining, the bone morphological changes are observed under the microscope.

#### 2.1.9 Bone Biomechanical Testing

The left femur specimen is removed from the normal saline in advance, placed on the biomechanical tester using forceps, and the span and loading speed are adjusted to 1.5 mm and 0.01 mm/s, respectively, until the femur finally breaks. The load-displacement curve recorded by the instrument calculates the maximum load, maximum deflection and stiffness.

### 2.2 Network Pharmacological Analysis

#### 2.2.1 Screening of Potential Active Ingredients and Targets of Drugs

To find the blood component of NRMDL, set ob>30% and OL > 0.18 in the Traditional Chinese Medicine Systems Pharmacology (TCMSP) database, screen the active ingredients of NRMDL, and combine sources from Pubmed, CNKI and other databases. Locate the corresponding target within the Traditional Chinese Medicine Systems Pharmacology (TCMSP) database. TCMSP database. After the resulting target is merged and deduplicated, the target is entered into the Uniprot database for the potential target of the blood component pulled into NRMDL. The species is selected as “*Homo sapiens*”, and the corresponding information is obtained, such as Uniprot ID, protein name.

#### 2.2.2 Screening for Disease Action Targets

The GeneCards database was used to search for related disease targets with “postmenopausal osteoporosis” as the keyword. The disease-related genes in the database were imported into an Excel table, and the overlapping gene entries were eliminated to obtain the disease-related action targets.

#### 2.2.3 Network Diagram of Drug-disease co-target Screening and Protein-related Effects

In the Venny2.1 online software mapping tool platform (https://bioinfogp.cnb.csic.es/tools/venny), the targets of NRMDL and PMOP were entered separately, and the Wayne map was drawn to obtain the common targets of drugs and diseases. The drug-disease common target was uploaded in the form of a gene symbol to the STRING database (https://string-db.org/) for retrieval. The species is set as “*Homo sapiens*”, the minimum required interaction score>0.9, hide the free protein, keep other parameters at default settings, and build a PPI network of protein interactions. Interactions were sorted according to the degree of association between proteins and the file saved in TSV format.

#### 2.2.4 Construction and Analysis of Network Models

Using Cytoscape 3.7.1 software, a “drug-ingredient-disease-target” interaction network diagram is constructed, with the active ingredient and effect of the drug represented by “nodes” and the interaction between nodes represented by “edges”. The main active ingredient of NRMDL was analyzed using the Network Analyzer function, and the key active ingredient of NRMDL anti-menopausal osteoporosis was screened by the degree value of the target in the network. The larger degree value signified more targets that interacted with the active ingredient and a more important role in anti-menopausal osteoporosis, which indicates the key component of NRMDL.

#### 2.2.5 Pathway Enrichment Analysis

Go function and KEGG pathway enrichment analysis of refined coronary heart tablets and co-opiac disease target proteins using DAVID database (https://david.ncifcrf.gov/). The first 20 entries of *p* < 0.05 were screened and visualized with the help of the WeChat website (http://www.bioinformatics.com.cn/) to illustrate the biological functions and related signaling pathways of NRMDL in the treatment of PMOP, and results were presented in the form of histogram and bubble chart.

#### 2.2.6 Molecular Docking Verification

NRMDL oral blood component was selected as the ligand and the target with a large medium value in the PPI network as the receptor. Protein structure of the key target was downloaded from the PDB database (https://www.rcsb.org/), and then imported into Autodock 4.2.6 software to dehydrate, hydrogenate, set the atomic type, and save it in pdbqt format. The 3D structure of the blood molecules was obtained from the Pubmed database (https://pubchem.ncbi.nlm.nih.gov/) and imported into Autodock 4.2.6 software for molecular docking. Autodock vina 1.1.2 was used to calculate binding energy, and the components with the highest binding energy were visualized using PyMol 2.3.5. software.

#### 2.2.7 Alkaline Phosphatase Staining, Alizarin Red Staining

Generation 3 cells were seeded at appropriate density in two 6-well plates and adhered with 10% drug-containing serum added to osteogenesis induction medium (50 μg/mL vitamin C + 10mmol/L sodium β-glycerophosphate + 10-8mol/L dexamethasone, preparation induction solution for induction). Intervention induced cells for 14 days, discarded cell culture medium, PBS washed once, 4% neutral paraformaldehyde fixation for 15 min, one of which was incubated with alkaline phosphatase staining solution at room temperature in the dark for 30 min, and washed with normal saline three times. Dry in the oven at 60°C, add alizarin red staining solution to evenly cover the cells, stain at room temperature for 30 min and wash well with distilled water. Observe and take pictures under an inverted microscope. The differences in alkaline phosphatase expression and mineralized nodules in each group were observed.

#### 2.2.8 Western Blotting Verification

Total protein was extracted from cells and tibia with RIPA lysis buffer with 1% protease and phosphatase inhibitors and 1% PMSF. Protein concentration was determined using the BCA Protein Kit (Biyotime, P0010S) assay. Samples were separated with 10% SDS-PAGE gel (ACE, WB0001) and transferred to PVDF membrane (Millipore, iseq00010) by wet transfer. Block for 2 h with 5% skim milk and then wash 4 times with TBST for 5 min each. PI3K (1:1000, Proteintech, 20584-1-AP), AKT (1:1000, Proteintech, 60203-2-Ig), RUNX2 (1:1000, Proteintech, AF6290), OCN (1:200, Proteintech, sc-81156), GAPDH (1:10000, Proteintech, 10494-1-AP), at 4°C overnight. The corresponding secondary antibody was added after washing (1:5000, Proteintech, 20536-1-AP; 20536-2-AP). Incubate at room temperature for 2 h and develop using ECL on the machine. Image J software was used to analyze the gray value of the image. The relative protein expression is determined by the gray value of the target protein/the gray value of the reference protein.

#### 2.2.9 Statistical Methods

Data were statistically analyzed using Graphad Prism. Normally distributed measurement data were expressed as the mean ± standard deviation, and univariate ANOVA was used to compare multiple groups to determine the statistical difference. *p* values <0.05 were considered statistically significant.

## 3 RESULTS

### 3.1 Effect of NRMDL on Serum Indices of Osteoporosis Rats

The serum from collected rats was detected with ELISA, and the results are shown in Fig. (**1A**). Compared with the Sham group, the serum level of TRPA in the OVX group increased (*p* < 0.01) and ALP level decreased (*p* > 0.05). The level of TRPA decreased in the NRMDL group compared with the OVX group, and Middle and High dose groups had significant statistical significance (*p* < 0.01, *p* < 0.01), and the ALP level increased but not statistically significant (*p* > 0.05). The levels of Zn, Ca, P and Na in serum were detected by trace element detector, and compared with the OVX group. Zn (*p* < 0.05), Ca (*p* > 0.05), P (*p* > 0.05), Na (*p* > 0.05) in the OVX group had a downward trend; compared with the OVX group, the content of Zn in serum (*p* < 0.01, *p* < 0.01) in Middle and High doses could be significantly increased in the NRMDL group compared with the OVX group. Na levels tended to rise without significant significance (*p* > 0.05).

The topologically related micro-CT data of bone microstructure are shown in Fig. (**1B**). The BMD of the group was significantly reduced compared with the Sham group (p<0.01); the NRMDL group and OT group showed increased BMD compared with the OVX group but were not statistically significant. In the topology-related data of bone microstructures, BV/TV (*p* < 0.01), Tb.N (*p* < 0.01) were significantly reduced in the OVX group compared with the Sham group. Tb.Sp (*p* < 0.001) increased significantly, compared with the OVX group, the NRMDL group and the OT group. BV/TV, Tb.N have an upward trend, and the Tb.Sp indicators all have a downward trend, but none are statistically significant. The Sham and OVX group did not have obvious cortical bone loss from the transverse and sagittal surface images, but the trabecular bone structure became thinner and the gap became larger. The trabecular bone is hardly visible from the 2D floor plan. In the NRMDL group and the OT group, the density of trabecular bone in the OVX group increased significantly, and the gap was relatively small. The NRMDL and OT groups prevent trabecular bone mineral loss caused by OVX.

### 3.2 Pathological Observation of the Femur

The results of pathological staining of bone histomy are shown in Fig. (**1C**). Trabeculae of rats in the Sham group are closely arranged, the structure is clear, the bone marrow cavity is small, and possess more active osteoblasts. The OVX group had obvious trabecular fracture, structural disorder, dense bone marrow cavity, and almost no active osteoblasts. Compared with the OVX group, the Middle, High and OT NRMDL groups had fewer trabecular fractures, tighter arrangement, fewer cavities, and a significant increase in active osteoblasts. The Middle, High and OT NRMDL groups significantly improved the pathological changes of trabeculae.

### 3.3 Bone Biomechanical Testing

The effect of NRMDL on bone mechanical properties is shown in Fig. (**1D**). After 12 weeks of administration, the maximum load (*p* < 0.01) and maximum stiffness (*p* < 0.05) of rats in the OVX group were significantly inhibited compared with the Sham group, but maximum displacement (*p* < 0.05). Compared with the OVX group, the maximum load of the femur (*p* < 0.01) was increased in all NRMDL groups. The maximum stress (*p* < 0.01, *p* < 0.01) was significantly increased in the Middle and High groups, and maximum displacement (*p* > 0.05) reduced in the Low and Middle groups. The OT group significantly improved maximum load, maximum stiffness, and maximum displacement.

### 3.4 Screening of the Active Ingredients and Disease Targets of NRMDL

**Fig. (2):**
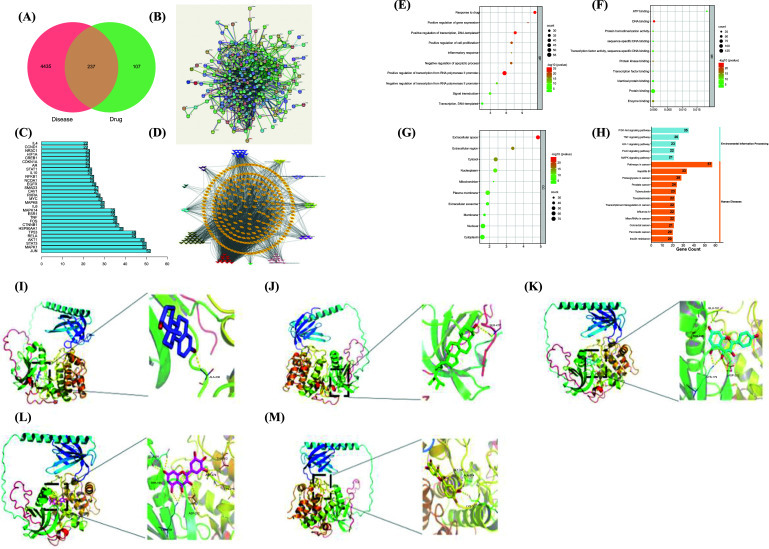
(**A**) Venn diagram: overlapping genes of NRMDL and PMOP; (**B**) STRING diagram: Gene interaction (PPI) network consisting of 238 overlapping genes. (**C**) Target ranking of the top 20 PPI networks. (**D**) Drug-compound-overlapping gene network: the red diamond represents the drug of NRMDL. (**E**) Biological processes (BP); (**F**) Cellular molecular function (MF); (**G**) Cell components (CC); (**H**) KEGG enrichment analysis (top 20). The abscissa represents the degree of enrichment. The smaller the FDR, the more important the concentration and the redder the color on the chart. The figure shows the 3D stereogram; (**I**) Estrogen and AKT, (**J**) β-sitosterol and AKT, (**K**) kaempferol and AKT, (**L**) myricetin and AKT, (**M**) quercetin and AKT.

Through the TSMSP database, 30% of OB ≥, 0.18% of DL ≥ were screened, and 107 active compounds were obtained, including 17 pomegranates, 20 piper Longum, 22 safflowers, 12 xanthanin, 10 cinnamon, 9 *Tribulus terrestris*, 7 mendon, 8 jade bamboo, 1 smallpox pollen, 6 winter sunflower fruits, and 5 nootropics (see Annex1). These active ingredients corresponded to 345 corresponding target genes. A total of 1143 PMOP-related genes were collected from the Genecard database; the target genes of NRMDL were compared with PMOP-related genes, and a total of 165 common target genes were collected using WeChat online production software (see Fig. **2A**).

### 3.5 PPI Network Construction and Analysis

Using STRING database, a potential target PPI network for the treatment of PMOP was constructed. The results were output in the form of pictures with 165 nodes, 878 sides, an average node number of 10.6, an average local aggregation coefficient of 0.443, and a PPI enrichment *p* value of 10^-17^ (Fig. **2B**). The top 30 enriched genes were calculated by R (Fig. **2C**).

### 3.6 The Results of the Construction and Analysis of the NRMDL-component-target Network

The NRMDL active ingredient and target information were imported into Cytoscape 3.7.2 software to build a “Chinese medicine-ingredient-target” network diagram (Fig. **2D**). In a network, the degree of a node represents the number of nodes in the network connected to the route; the larger degree values, the more likely the compound is to function. It can be concluded from network topology that the top 8 degree values in the network are quercetin, kaempferol, β-sitosterol,β-estradiol, Myricetin. Therefore, we speculate that the above ingredients may be important components of NRMDL in the treatment of PMOP.

### 3.7 GO Feature Enrichment Analysis

In the DAVID database, GO function enrichment analysis was performed on the common target genes of NRMDL and PMOP. The enrichment results show that the biological process of NRMDL treatment of PMOP is mainly related to the positive transduction of intracellular signals, cell-to-drug stimulation, cell proliferation, negative regulation of apoptosis process, and negative regulation of transcription of RNA polymerase initiator. Molecular functions are mainly concentrated in protein binding, enzyme binding, DNA binding, transcription factor activity and binding, sequence-specific DNA binding, ATP protein binding, cell components mainly involve nucleus, cytoplasm, plasma membrane, extracellular space, plasma membrane, mitochondria, nuclear plasma, etc. The analysis results showed that PMOP involves multiple biological processes *in vivo*, and NRMDL may play a therapeutic role by regulating these biological processes. The top 10 were taken for Visually display data based on count rankings using bubble charts. According to the count ranking and *p* < 0.05 (Fig. **2E**-**G**).

### 3.8 KEGG Enrichment Analysis

KeGG pathway enrichment analysis was performed on the common target genes of NRMDL and PMOP, and a histogram was made with the top 20 KEGG pathways in descending order (Fig. **2H**). The abscissa coordinate represents the number of enrichments, and the ordinate coordinate represents the pathway and disease type. The results of KEGG enrichment show that the pathways are mainly distributed in the PI3K-Akt, HIF-1, FoxO, MAPK, and TNF signal pathways.

### 3.9 Molecular Docking

Using Autodock 4.2.6, quercetin, kaempferol, β-sitosterol, apigenin, arbutyl alcohol, β-estradiol in the NRMDL active compounds were connected to the active pockets of AKT, IL1B, IL6, INS, JUN, STAT3, TNF, TP53 (the top 8 genes in the degree score in the PPI network map and cytoscape software), respectively. Vina score (Table **2**) is the use of Autodock vina software with the corresponding pocket parameters of the ligand and receptor binding complex score. Lower scores represent greater binding energy, and higher affinity, and the best target gene AKT binds to the 5 compounds. Using PyMol 2.3.5. software for visual display, molecular docking mode diagram is shown in Fig. (**2I**-**M**).

### 3.10 ALP Staining *Versus* Mineralized Staining

**Fig. (3):**
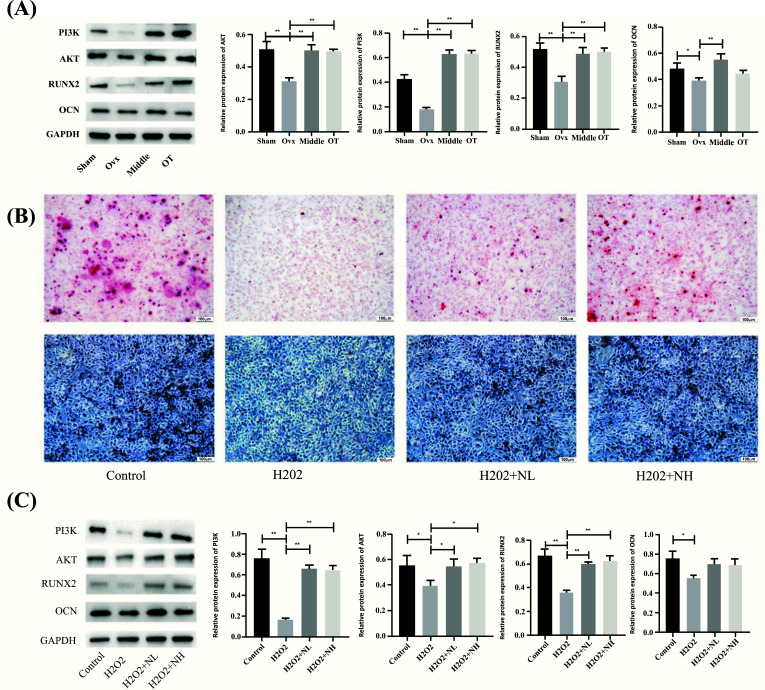
(**A**) Effects of NRMDL on the expression of PI3K, AKT, RUNX2 and OCN proteins in OVX rats. **B**) Red is mineralized staining and blue-violet is ALP staining. **C**) Effects of NRMDL on the expression of PI3K, AKT, RUNX2 and OCN proteins in osteoblasts. *p* < 0.05, **p < 0.01.

To understand the effect of NRMDL on osteoblastic osteogenesis, ALP staining and alizarin red staining were performed for 7 and 14 days, respectively. Positive areas of ALP and alizarin red staining in the H_2_O_2_ group were significantly lower than those in the Control group, while the positive rates of ALP staining and alizarin red in the low-dose NRMDL (NL) and high-dose NRMDL (NH) groups were significantly higher than those in the H2O2 group. Positive rates of the low-dose group were higher than those in the high-dose group (Fig. **3B**).

### 3.11 Western Blotting

Based on bone density and pathological detection, we found that the high-dose group of NRMDL had the best effect. Protein changes of the high-dose group were mainly verified in animal experiments, and results showed that levels of PI3K, AKT, RUNX2 and OCN protein in the OVX group were significantly reduced compared with the sham group, while the NRMDL group and OT group could improve this situation. Compared with the OVX group, the upward trend of NRMDL group was statistically significant, while the changes of PI3K, AKT, RUNX2 in the OT and NRMDL group were similar and the differences were statistically significant, while the OCN protein had an upward trend but was not statistically significant (Figure **3A**). The changes of PI3K, AKT, RUNX2 and OCN in osteoblasts were detected after drug-containing serum was added to cells. After H_2_O_2_ intervention, the above proteins decreased significantly. Low and high doses of NRMDL had a reversal effect on the above proteins, and the differences were statistically significant, among which the OCN protein changes were not statistically significant (Fig. **3A**-**C**) [3-12].

## 4 DISCUSSION

The increase in bone fragility caused by PMOP is the leading cause of fractures in older women globally. Due to its complex pathological mechanism, there are certain deficiencies in effective treatments [13]. Mongolian medicine has received increasing attention in its efficacy as part of ancient Chinese traditional medicine [14]. A classic Mongolian prescription, NRMDL, has great clinical efficacy and is widely known among Inner Mongolian patients. For the purpose of promoting Mongolian medicine and benefiting patients with PMOP, we conducted relevant experimental verification. This study successfully established a de-ovulatory rat model in which estrogen levels suddenly decreased. There was an increase in osteoclast formation while osteoblast formation decreased in this case. The presence of bone switching results in increased bone resorption within the body, increasing the risk of fractures in the trabecular bone. As reported in this study, NRMDL treatment decreased bone resorption in OVX rats, increased bone formation, and inhibited bone loss. Micro-CT results showed that the NRMDL treatment group could significantly improve the bone trabecular density of rats in the OVX model, consistent with HE staining results. Furthermore, we performed biomechanical tests and found that NRMDL improved bone strength in ovary-de-ovulatory rats, further demonstrating NRMDL efficacy in treating PMOP. The specific mechanism, however, is unknown. Thus, network pharmacology and molecular docking are used to predict the potential biological basis for treating PMOP.

A network pharmacology approach focuses on understanding the complex relationships between compounds, targets, diseases, and biological systems [15]. The TCM theory promotes holistic and systematic thinking [16]. This paper presents a preliminary investigation into the complex molecular mechanism of NRMDL's pharmacological effect on PMOP using molecular docking technology and network pharmacology. It is currently possible to study the pharmacological effects, safety, and complex molecular mechanisms of traditional Chinese medicines based on network pharmacology. In addition to validating the results of network pharmacology, computer simulation docking techniques also examines whether overlapping proteins and major active ingredients behave similarly in NRMDL. As a result of the screening of active ingredients and the analysis of compound target networks, it is believed that quercetin, kaempferol, β-sitosterol, and arbutin may be the main components of NRMDL having pharmacological effects on PMOP. This network's most moderately valued flavonoid is quercetin, widely found in a variety of herbs, and exerts a variety of pharmacological effect such as anti-inflammatory, anti-cancer, anti-aging, anti-coagulation and regulation of endocrine among other effects [17-19]. It has been found that quercetin activates the AMPK/SIRT1 signaling pathway to promote osteogenesis in mouse BMSCs [20]. Kaempferol-tong is an anti-osteoporosis antioxidant that also regulates mitochondria [21]. It has also been shown that β-sitosterol reduces glucocorticoid-induced bone loss in rats by inhibiting RANKL/OPG signaling [22]. It is demonstrated *in vivo* that arbutin improves bone pathology and promotes differentiation and proliferation of osteogenesis precursor cells *in vitro* through the ERK pathway [23].

In addition, network pharmacology algorithms are used to screen out key proteins, for understanding the key targets and pathways of the NRMDL treatment of PMOP. AKT is considered a key protein, and crucial to bone formation. It has been found that AKT acts both as a negative regulator of osteoblast differentiation and as a powerful positive regulator of osteoblast coupling to osteoblast production [24]. A study by Kawamura *et al.* found that mice lacking Akt1 developed osteopenia, and that osteoclasts lacking Akt1 displayed impaired bone resorption because of impaired cell autonomy [25]. A knockout mouse lacking Akt1 has a reduced body size, shortened bone length, and delayed secondary ossification. Akt1/Akt2 double knockout mice show dwarfism and skeletal ossification developmental delays shortly after birth. Furthermore, the KEGG analysis reveals the importance of the PI3K-Akt signal path [26]. It may be one of the key mechanisms through which NRMDL exerts its effectiveness [27]. There are a number of pathological conditions associated with the PI3K/Akt pathway, including osteoporosis, osteoarthritis, and osteosarcoma. Among its functions are to stimulate osteoclasts and osteoblasts to divide, differentiate, and undergo apoptosis [28, 29]. Based on a study by Ma *et al.* [30], it appears that the IGF-activated PI3K/Akt signal transduction pathway modulates differentiation of osteoblasts by TRPV6. PI3K/AKT/mTOR/S6K1 (S6 kinase 1) signaling pathways have been implicated in the promotion of osteoblast survival, differentiation, and migration through TNF-β1 [31].

Traditional Mongolian medicine is used in Chinese ethnic minority areas; Mongolians are known for their continuous practice and innovation. As a result, Mongolian medicine has maintained its vitality over the centuries. Throughout its history, Mongolian medicine has demonstrated strong vitality, and as a result has not been replaced by other medical schools, but has developed its own unique medical system. The language barrier and traffic congestion limit the interpretation of Mongolian medicine using molecular experiments, and the biological basis of its treatment of disease is often unknown. It is possible to predict some mechanisms underlying NRMDL using network pharmacology, but there are some limitations. In the first instance, active ingredients are identified through the database, which is not experimentally validated and may deviate from main acting ingredients. Furthermore, molecular docking and KEGG predicted that most targets passed, and did not verify their results from protein levels and cellular models.

## CONCLUSION

Altogether, our findings suggest it may be possible to improve OVX-induced bone loss by using NRMDL to reduce osteoclast activity and this statement elucidates the augmentation of osteoblast functionality. Osteoblast function. Furthermore, it has been found that NRMDL in the treatment of PMOP works by targeting key compounds and proteins such as quercetin, kaempferol, and AKT. The PI3K-Akt signaling pathway is also involved in therapeutic mechanisms. As a consequence, this study provides a plausible experimental base for treating postmenopausal osteoporosis with NRMDL and explores its mechanism of action in a preliminary manner.

## Figures and Tables

**Fig. (1):**
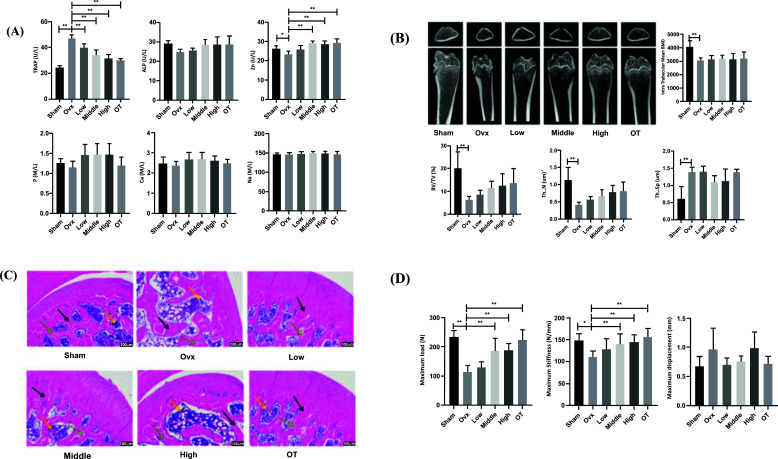
(**A**) Effect of NRMDL on serum markers in OVX rats. Tartaric acid phosphatase (TRPA), alkaline phosphatase (ALP), zinc (Zn), calcium (Ca), E phosphorus (P), sodium F (Na). The data is displayed as an average ± SD (n=8 only/group); (**B**) The micro-CT scan of the distal right femur showed the typical plain scan image (sagittal plane and transverse plane) and parameters of the distal femur; (**C**) NRMDL promoted osteogenesis in rats, and HE staining was performed after treatment. The green arrow refers to active osteoblasts, the black arrow refers to the trabeculae, and the yellow arrow refers to the bone marrow cavity. (**D**) Effect of NRMDL on skeletal strength of right skeletal in OVX rats. Maximum stiffness; Maximum displacement; Maximum load; Results are shown as mean ±SD. * *p* < 0.05, ** *p* < 0.01.

**Table 1:** Composition of NRMDL.

**Pharmaceutical Name**	**Botanical or Zoological Name**	**Chinese**	**Content(g)**
Punica Granatum	*Punica granatum* Linn	Shi Liu	30
Alpinia Oxyphylla	*Alpinia oxyphylla* Miq	Yi Zhi Ren	15
Polygonatum Odoratum	*Polygonatum odoratum* (Mill.)	Yu Zhu	12
Piper Longum	*Piper longum* L.	Bi Ba	12
Polygonatum Kingianum	*Polygonatum sibiricum* Redouté	Huang Jing	9
Asparagus Cochinchinensis	*Asparagus cochinchinensis* (Lour)	Tian Men Dong	9
Tribulus Terrestris	*Tribulus terrestris* L	Ji Li	9
Radix Trichosanthis	*Trichosanthes kirilowii* Maxim	Tian Hua Fen	9
Carthamus Tinctorius	*Carthamus tinctorius* Linn	Hong Hu	9
Althaea Rosea	*Althaea rosea* (Linn.) Cavan	Shu Kui	9
Althaea Rosea	*Cinnamomum cassia* Nees ex Blume	Rou Gui	3

**Table 2:** Vina score.

**Molecular**	**Molecular Structure**	**Protein**	**Affinity (kcal/mol)**
Estradiol	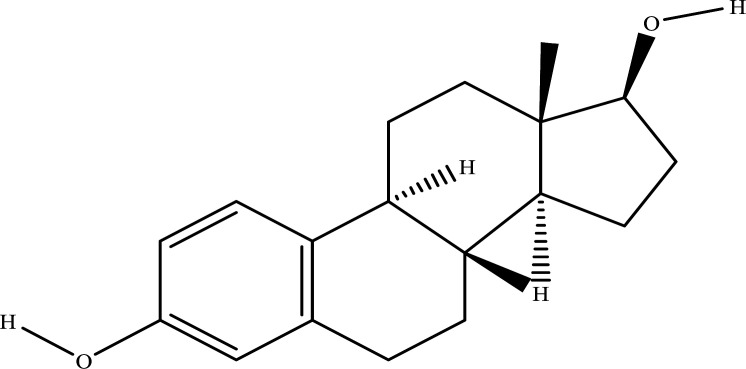	AKT1 IL1β IL6 INS JUN STAT3 TNF P53	-8.6 -7.3 -6.2 -7.7 -6.0 -7.4 -7.6 -7.4
Sitosterol	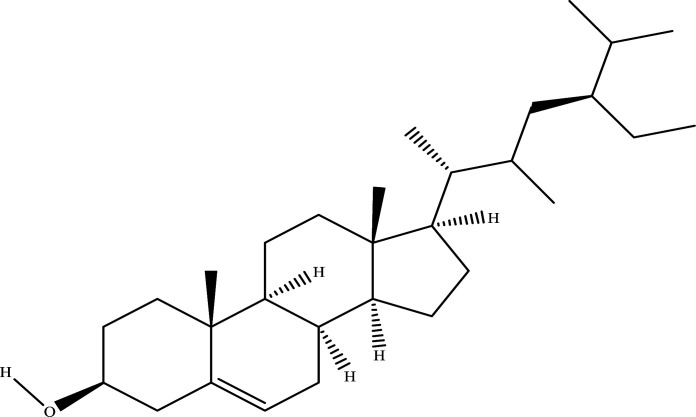	AKT1 IL1β IL6 INS JUN STAT3 TNF P53	-7.9 -7.8 -6.7 -7.6 -5.9 -7.8 -6.7 -7.4
Quercetin	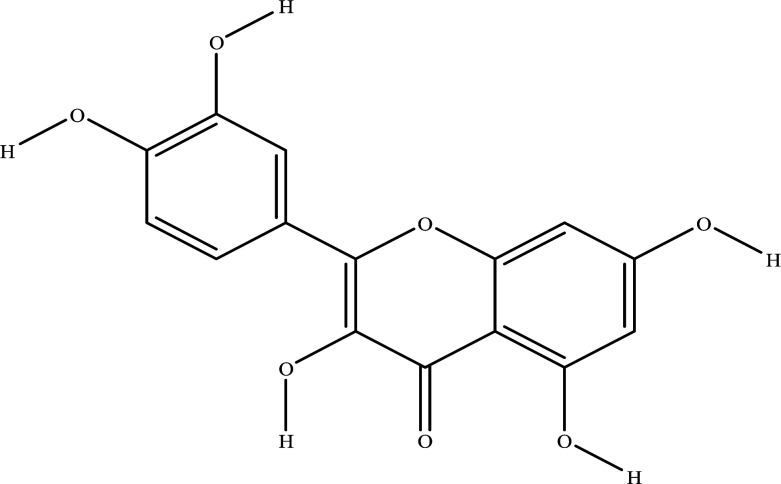	AKT1 IL1β IL6 INS JUN STAT3 TNF P53	-7.3 -6.9 -6.5 -6.2 -5.3 -6.9 -6.1 -7.6
Myricetin	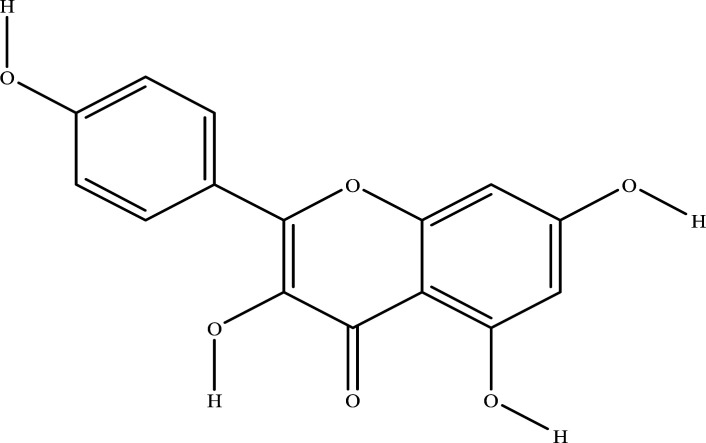	AKT1 IL1β IL6 INS JUN STAT3 TNF P53	-8.2 -7.2 -7.6 -6.3 -5.8 -8.0 -6.3 -7.7
Kaempferol	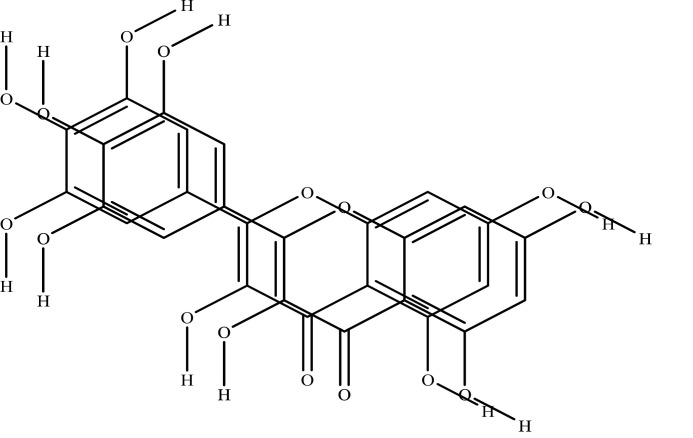	AKT1 IL1β IL6 INS JUN STAT3 TNF P53	-8.0 -6.6 -7.2 -6.1 -5.4 -7.4 -6.2 -7.5

## Data Availability

The data and supportive information is available within the article.

## LIST OF ABBREVIATIONS

BP: Biological Processes
CC: Cell Components
NRMDL: Mongolian Medicine Narenmandula
PMOP: Postmenopausal Osteoporosis
TCMSP: Traditional Chinese Medicine Systems Pharmacology
